# Surge: a fast open-source chemical graph generator

**DOI:** 10.1186/s13321-022-00604-9

**Published:** 2022-04-23

**Authors:** Brendan D. McKay, Mehmet Aziz Yirik, Christoph Steinbeck

**Affiliations:** 1 School of Computing, Australian National University, Canberra, ACT 2601 Australia; 2 Institute of Inorganic and Analytical Chemistry, Friedrich-Schiller-University, Lessingstr. 8, 07743 Jena, Germany

**Keywords:** Structure generation, Constitutional isomers, Canonical generation path

## Abstract

Chemical structure generators are used in cheminformatics to produce or enumerate virtual molecules based on a set of boundary conditions. The result can then be tested for properties of interest, such as adherence to measured data or for their suitability as drugs. The starting point can be a potentially fuzzy set of fragments or a molecular formula. In the latter case, the generator produces the set of constitutional isomers of the given input formula. Here we present the novel constitutional isomer generator surge based on the canonical generation path method. Surge uses the nauty package to compute automorphism groups of graphs. We outline the working principles of surge and present benchmarking results which show that surge is currently the fastest structure generator. Surge is available under a liberal open-source license.

## Introduction

Chemical structure generators enumerate or generate molecular graphs of organic or bioorganic molecules. They are an integral part of systems for computer-assisted structure elucidation (CASE) [1] and can be used to create molecular libraries for virtual screening [2, 3] or enumerate chemical spaces in general [4]. The history of chemical graph generators goes back at least to the 1960s DENDRAL project which was aimed at the CASE of organic molecules based on mass spectrometric data [5]. DENDRAL was developed for NASA’s Mariner program to search for life on Mars [5, 6]. Its structure generator used substructures as building blocks and was able to deal with overlapping substructures. In the early history of the structure generators, ASSEMBLE was another building block based structure generator [7]. In the field, there is a family of generators based on mathematical theorems such as algorithmic group theory [8] and combinatorics [9]. Besides DENDRAL, MASS [10] was also another good example for the applications of mathematical theorems in structure generation. It was a tool for the mathematical analysis of molecular structures. SMOG [11] was the successor of the MASS algorithm.

We have recently reviewed the history of chemical graph generators in detail [12].

While most structure generators work in a deterministic way, i.e. exhaustively generate structures according to given boundary conditions [13], stochastic generators were also suggested for large molecular spaces [14].

Among the currently available structure generators, such as DENDRAL, ASSEMBLE, SMOG, COCON [15] and LSD [16], MOLGEN [17] constituted the state-of-the-art for decades in terms of speed, completeness and reliability.

The first version of MOLGEN was based on the strategy of DENDRAL software and developed to overcome the limitations of DENDRAL [18]. The software is based on the orderly graph generation method [19]. Although MOLGEN is the de facto gold standard in the field, it has the downside of being closed-source software. The algorithm cannot be further developed or modified by scientists based on their interests. The most efficient and fast open-source chemical graph generator was MAYGEN [20] based on the orderly generation method. However, MAYGEN is approximately 3 times slower than MOLGEN.

The state of the art of large scale structure generation was recently set by the lab of Jean-Louis Reymond [21] in developing an in-house solution for a nauty-based structure generator, which enabled them to produce the numeration of 166 billion organic small molecules in the chemical universe database GDB-17. To the best of our knowledge, this in-house generator was not released as open-source or otherwise.

Thus, there is still the need for an efficient open-source chemical graph generator. In [20] we expressed the hope to “trigger a surge in the development of improved and faster” structure generators. Here we present the novel structure generator surge, based on the principle of the canonical generation path method. Surge is open-source and outperforms MOLGEN 5.0 by orders of magnitude in speed. Furthermore, surge is easily extensible with more features and adaptable to further application.

## Implementation

### Data

We assembled a list of molecular formulae for benchmarking surge against MOLGEN 5.0 in Tables 1, 2. These formulae were taken from the natural products database COCONUT [22]. The size of these molecular formulae varies and is enough to challenge even the best constitutional isomer generators available (see Results section).

**Table 1:** Execution time (seconds) for selected MF of natural products on a compute-optimized c2-standard-4 Google cloud VM

Name of notable isomer	Molecular formula	Species	#Isomers	SURGE time (s)	MOLGEN time (s)
Bassianolone	C_10_H_16_O_5_	*Beauveria bassiana*	1,092,378,303	69	5146
Pantothenate	C_9_H_17_NO_5_	*Arabidopsis thaliana*	1,652,346,465	165	11,122
Lysopine	C_9_H_18_N_2_O_4_	*Parthenocissus tricuspidata*	5,979,199,394	289	27,250
Cribronic acid	C_6_H_11_NO_7_S	*Cribrochalina olemda*	2,375,932,807	323	13,445
Antibiotic CV-1	C_7_H_14_N_2_O_6_	*Streptomyces CO-1*	4,193,416,397	448	24,030
Thr-Thr	C_8_H_16_N_2_O_5_	*Trypanosoma brucei*	5,955,022,220	575	37,103
*O*-Succinyl-l-Homoserine	C_8_H_13_NO_6_	*Escherichia coli K12*	5,639,328,954	629	35,128
Etrogol	C_13_H_18_O_2_	*Stachylidium*	6,316,260,274	746	44,395
Indoleacetamide	C_10_H_10_N_2_O	*Pseudomonas savastanoi*	13,290,477,420	1187	59,910
Colletotricole A	C_9_H_13_NO_3_S	*Colletotrichum gloeosporioides A12*	20,902,484,656	1765	88,151
Nigerapyrone E	C_11_H_12_O_4_	*Aspergillusniger MA-132*	31,627,481,929	2179	181,725
Siastatin B	C_8_H_14_N_2_O_5_	*Streptomyces verticillus var. quintum*	27,692,853,176	2628	183,167
P-Hydroxyhippuric acid	C_9_H_9_NO_4_	*Homo sapiens*	21,964,168,804	2731	121,362
Deacetyldemethylanisomycin	C_11_H_15_NO_3_	*Streptomyces sp. strain SA3097*	95,541,477,841	4229	580,772
Isoleucylisoleucyl anhydride	C_12_H_22_N_2_O_2_	*Cordyceps bassiana*	59,576,199,503	4782	516,950
Hydantocidin	C_7_H_10_N_2_O_6_	*Streptomyces hygroscopicus*	40,946,033,849	5238	262,323
Aerugine	C_10_H_11_NO_2_S	*Pseudomonas aeruginosa*	93,330,898,027	8124	533,440
Flavensomycinoic acid	C_9_H_9_NO_5_	N/A	113,165,341,837	8870	793,389
Dopamine 4-*O*-Sulfate	C_8_H_11_NO_5_S	*Homo sapiens*	89,694,168,554	9880	606,333
Pestalactam C	C_10_H_10_ClNO_3_	*pestalotiopsis *sp*.*	232,824,605,597	14,830	1,700,022
Glugaba	C_9_H_16_N_2_O_5_	*Escherichia coli*	176,162,377,006	16,265	1,315,301
Shihunine	C_12_H_13_NO_2_	*Dendrobium loddigesii*	427,207,647,324	19,769	2,504,164
Gostatin	C_8_H_10_N_2_O_5_	*sumanensis*	187,389,585,693	21,781	1,422,863
Elaiomycin	C_13_H_26_N_2_O_3_	N/A	303,023,674,167	29,288	2,729,280
Oryzoxymycin	C_10_H_13_NO_5_	*Streptomyces venezuelae var. oryzoxymyceticus*	552,024,644,350	54,372	6,325,646
Gammaglucys	C_8_H_14_N_2_O_5_S	*Mus musculus*	699,785,343,381	69,844	4,989,287
Phyllurine	C_10_H_10_N_2_O_3_	*Phyllanthus urinaria*	1,511,861,775,412	83,186	8,292,585
Vanilloylglycine	C_10_H_11_NO_5_	*Homo sapiens*	1,182,104,108,010	133,136	21,426,660
Deoxyuridine	C_9_H_12_N_2_O_5_	*Phakellia mauritiana*	1,795,817,811,706	180,727	13,983,652
Sulphostin	C_5_H_13_N_4_O_5_PS	N/A	2,029,911,211,739	226,830	11,893,149

Times for MOLGEN 5.0 were determined with the -noaromaticity flag to achieve comparability. Both MOLGEN and surge were instructed to generate but not to output structures. Both generators generated the same number of isomers

**Table 2:** Execution time (seconds) for selected MF of natural products on a compute-optimized c2-standard-4 Google cloud VM

Molecular formula	-p0:1		-P		-B5		-B9	
	#Iso	Time	#Iso	Time	#Iso	Time	#Iso	Time
C_11_H_19_N_3_O	58,175,540,999	3746	72,486,967,073	5046	69,648,876,936	4978	51,275,365,737	3048
C_11_H_18_N_2_O_2_	53,925,725,334	3648	67,177,819,545	4914	64,367,528,959	4838	47,278,714,772	2946
C_11_H_15_NO_3_	64,661,412,269	4759	94,361,334,994	7682	89,131,725,467	7512	54,627,135,057	3595
C_9_H_18_N_2_O_4_	5,810,409,623	519	5,979,199,394	541	5,918,503,858	538	5,583,717,596	484
C_11_H_12_O_4_	17,216,498,094	1894	30,438,650,047	4485	28,660,902,856	3777	14,044,693,099	1256
C_10_H_16_O_5_	989,273,530	107	1,092,378,303	122	1,060,206,152	122	895,109,814	88
C_13_H_20_O_2_	1,211,481,307	147	1,514,909,702	203	1,443,691,541	197	1,038,843,543	101
C_8_H_11_NO_6_	12,795,251,232	1511	15,771,433,061	1953	15,035,794,185	1942	11,169,581,507	1217
C_9_H_9_NO_5_	62,471,125,788	8244	109,135,601,623	16,008	102,826,808,386	15,645	51,607,646,947	6062
C_12_H_13_NO_2_	177,274,446,997	13,639	382,246,449,331	34,476	381,333,513,411	34,285	147,423,365,942	9700

Surge was run with its options and instructed to generate but not to output structures

### Algorithm and mathematical background

Surge is based on the nauty [23] package for computing automorphism groups of graphs as well as canonical labels. Like nauty, surge is written in a portable subset of C and runs on a considerable number of different systems.

**Fig. 1:**
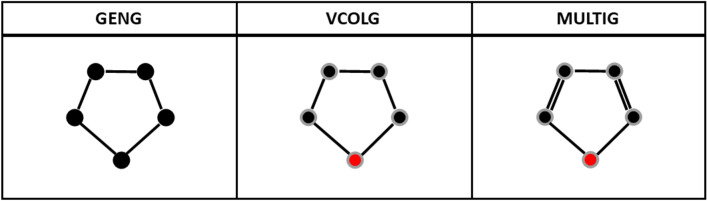
An example case for the combination of geng, vcolg and multig functions for the furan molecule, C_4_H_4_O. First the simple graph is constructed. The nodes are coloured as, black for carbons and red for the oxygen. In multig, the edge multiplicities are optionally increased to create multiple bonds

Surge is an integration of three existing tools from the nauty suite [24]: (a) geng generates simple graphs based on certain boundary conditions, (b) vcolg colors vertices in the output of geng and (c) multig inserts multi-edges in the output of the first two tools (Fig. 1).

**Fig. 2:**
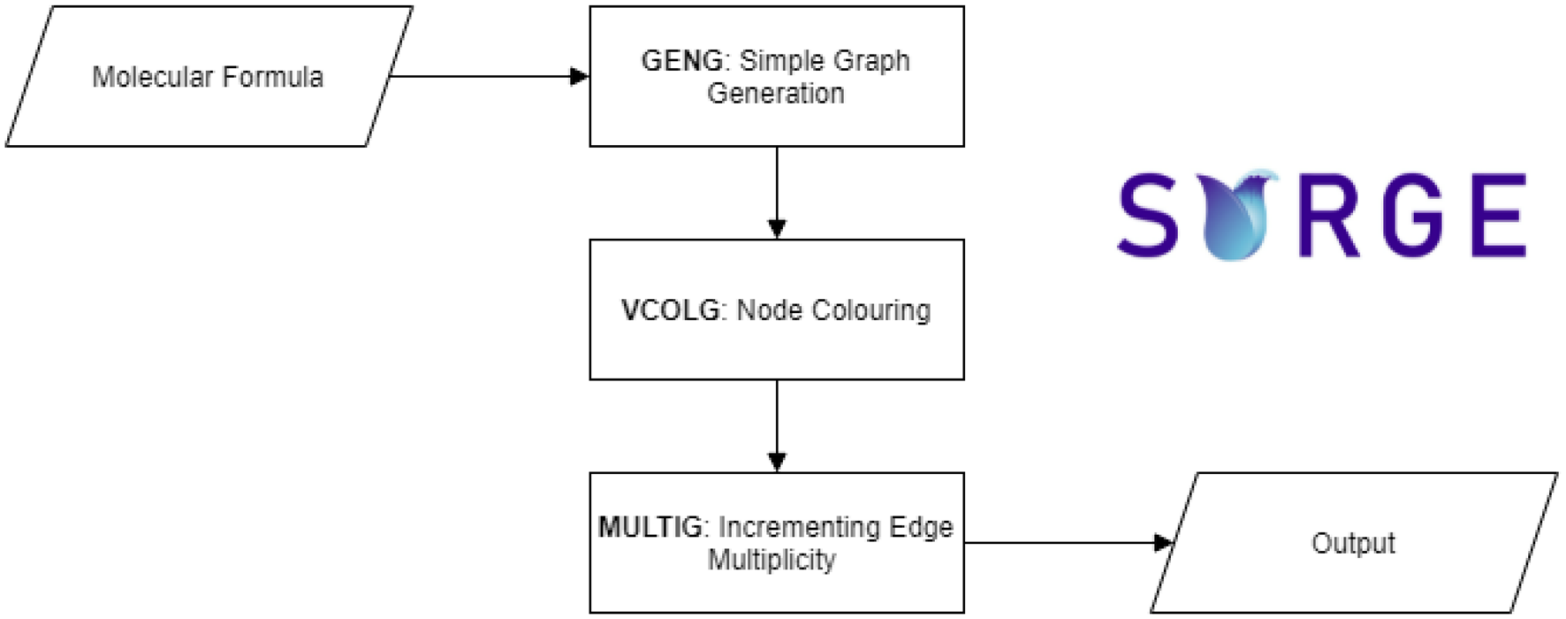
Surge flowchart

An *isomorphism* between two graphs is a bijection between their vertex sets that maps edges onto edges. If the graphs have adornments, such as atom types for the vertices or bond multiplicities for the edges, then those adornments must be preserved by the mapping. If the two graphs are the same; i.e., the isomorphism is from a graph to itself, it is called an *automorphism*. The automorphisms form a group under the operation of function composition, called the automorphism group (Fig. 2).

The meanings of isomorphism and automorphism are different for each of the three stages in our algorithm. Referring to Fig. 1, at the first stage (which we call a simple graph) there are no vertex or edge adornments and all rotations and reflections, 10 in total, are automorphisms. When vertex adornments are added in the second stage, the atom type becomes significant so only the identity mapping and the reflection through the oxygen atom are automorphisms. In the final stage, edge adornments are added but in this example the automorphism group is not further reduced since the reflection through the oxygen atom preserves both atom type and bond multiplicity. Note how the automorphism groups in the second and third stages are subgroups of the automorphism groups in the previous stages.

### First stage

Input to surge consists of a molecular formula such as C_7_H_12_O_2_S. Based on the element counts, in this case C = 7, O = 2, S = 1, H = 12, the atom valencies are used to calculate the plausible range of the number of edges of a connected simple graph representing the topology of a molecule with this formula, with hydrogen atoms omitted. Then geng is called to generate all the connected simple graphs with those parameters, subject also to a maximum degree condition depending on the molecular formula [25]. Geng generates one graph from each isomorphism class and these are passed to the second stage as they are produced, without any need to store them [25]. In this example, there are 10 non-hydrogen atoms and the number of edges is in the range 9–11.

### Second stage

**Fig. 3:**
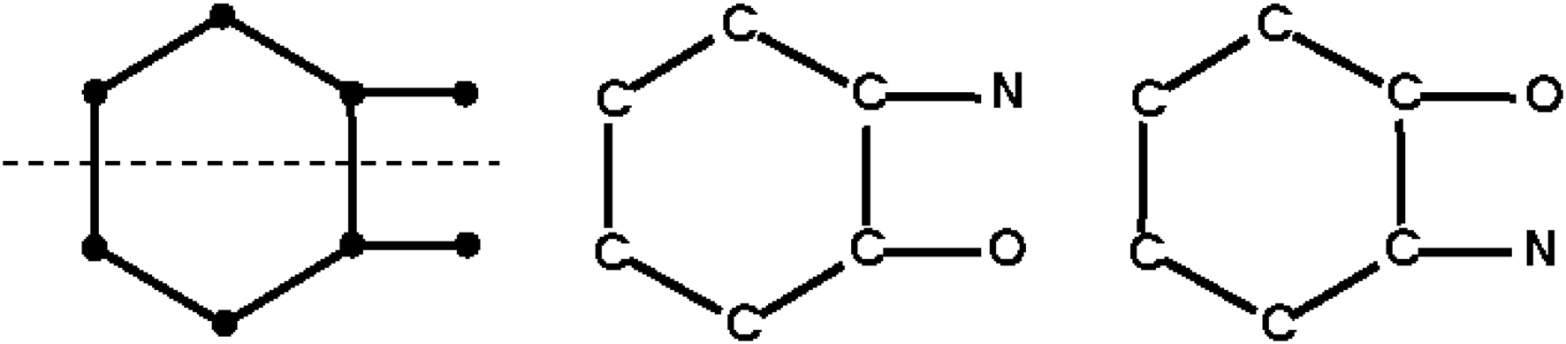
The simple graph on the left has an automorphism which is a reflection about the dashed line. This shows that the second and third images are equivalent and so will lead to the same molecular structures when bond multiplicities are assigned. So we only want to keep one of them

Given a simple graph G from the first stage, the second stage assigns elements to vertices in all distinct ways. The element counts must be correct, and we must have valence $\ge$ degree at each vertex. More onerously, we only want one member of each equivalence class of element assignment under the automorphism group of G (Fig. 3). We next explain how this is accomplished.

The vertices of G are arbitrarily numbered 1,2,…,n. An element assignment can be represented as a list showing the element assigned to each vertex in order of vertex number. For example, a valid list might be L = (C,C,C,S,O,C,C,C,O,C).

Automorphisms of G have an action on lists that permutes their entries. Namely, for list L and automorphism $\gamma ,$ the list $\gamma$(L) assigns the same element to vertex $\gamma$(v) as L assigns to v, for each vertex v. Thus,

$$\mathrm{L }= \left(\mathrm{C},\mathrm{C},\mathrm{O},\mathrm{S},\mathrm{O},\mathrm{C},\mathrm{C},\mathrm{C},\mathrm{C},\mathrm{C}\right) \, \mathrm{and }\, \gamma = \left(1 2 3\right)\left(5 6\right)\mathrm{ imply } \gamma (\mathrm{L}) = (\mathrm{O},\mathrm{C},\mathrm{C},\mathrm{S},\mathrm{C},\mathrm{O},\mathrm{C},\mathrm{C},\mathrm{C},\mathrm{C}).$$

If L is a list of elements and $\gamma$ is an automorphism, L and $\gamma$(L) give equivalent assignment of elements to the vertices of G. Our task in this stage is to choose exactly one assignment from each equivalence class. Given a fixed ordering of the elements, for example C < O < S, two lists can be compared lexicographically, for example

$$(\mathrm{C},\mathrm{C},\mathrm{C},\mathrm{S},\mathrm{O},\mathrm{C},\mathrm{C},\mathrm{C},\mathrm{O},\mathrm{C}) < (\mathrm{C},\mathrm{C},\mathrm{O},\mathrm{C},\mathrm{S},\mathrm{C},\mathrm{C},\mathrm{O},\mathrm{C},\mathrm{C})$$

This enables us to define

$$\mathrm{canon}(\mathrm{L}) =\mathrm{ max }\{ \gamma (\mathrm{L})\, |\, \gamma \, \mathrm{ in\, Aut}(\mathrm{G}) \},$$

the maximum list in the equivalence class of L. Note that canon(L) = canon(L’) if L and L’ are equivalent, so there is a unique maximum list L* in the equivalence class and we can recognize it by the condition canon(L*) = L*. To put it another way, if $\gamma$(L) > L for any automorphism $\gamma$ then L $\ne$ L*; otherwise L = L*.

Now we describe the conceptual method for the second stage. For given G:
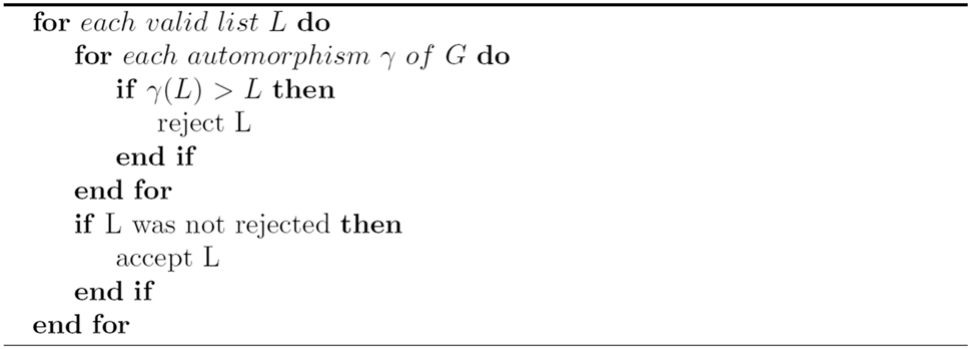


This algorithm is efficient if the automorphism group Aut(G) is small, but that is not always the case. Therefore, we adopt a more complex approach. An automorphism of G is called *minor* if it merely swaps two leaves (vertices of degree 1) that have a common neighbour. The minor subgroup M $\le$ Aut(G) is the subgroup generated by all the minor automorphisms.

**Fig. 4:**
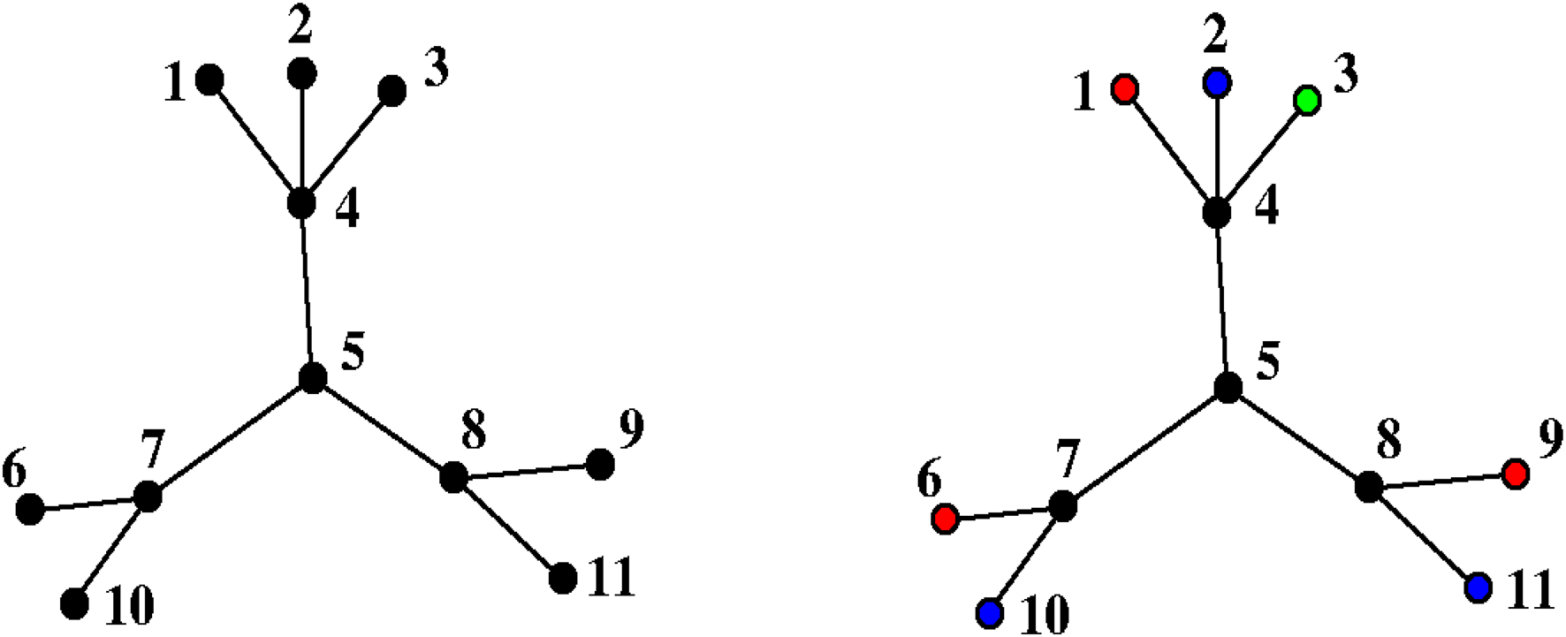
A graph with 3 flowers and the colouring used to compute N

A *flower* is a maximal set of leaves with the same neighbour. In the left graph of Fig. 4, the flowers are {1,2,3}, {6,10} and {9,11}. The minor subgroup M consists of all automorphisms that preserve the flowers, such as (1 2 3)(9 11). The order of M is $3!\times 2!\times 2! = 24$. In addition to M, the automorphism group may contain automorphisms that do not preserve the flowers, such as (6 11)(7 8)(9 10). To capture such automorphisms, we colour the graph as in the right side of Fig. 4. Vertices not in flowers are coloured black. Within each flower, vertices are coloured red, blue, green, … in order of vertex number, using a fixed list of colours that does not include black. Now let N be the group of automorphisms that respect the vertex colours. In the example, N has only the identity and (6 9)(7 8)(10 11).

An arbitrary automorphism of G can be obtained by first applying an element of N to capture how the flowers are mapped to each other, and then applying an element of M to capture the movement of leaves within each flower. In both steps the choice is unique, so we have a factorization

$$\mathrm{Aut}(\mathrm{G}) =\mathrm{ NM }= \{ \gamma \delta \, | \, \gamma \, \mathrm{ in\, N},\, \delta \,\mathrm{ in \, M }\}.$$

(In the language of group theory, M is a normal subgroup and N is a complete set of coset representatives.) In the example, consider (1 2)(6 11)(7 8)(9 10). It swaps the flowers {6,10} and {9,11} so we choose the element of N which does that, namely $\gamma$ = (6 9)(7 8)(10 11). Then we have to arrange the leaves within the flowers with an element of M, namely $\delta$ =(1 2)(6 10)(9 11). This achieves $\gamma \delta$ = (1 2)(6 11)(7 8)(9 10).

The main advantage of factoring Aut(G) = NM is the following.

#### **Theorem**

*For any list L, L = canon(L) if and only if L = max { *$\delta$*(L) | *$\delta$ *in M} and L = max { *$\gamma$*(L) | *$\gamma$ *in N}*.

#### *Proof*

The “only if” direction is obvious since M and N are subsets of Aut(G). Suppose in the other direction that L = max {$\delta$(L) | $\delta$ in M} and L = max {$\gamma$(L) | $\gamma$ in N}. From the factorization of Aut(G) we know that L* = $\delta$($\gamma$(L)) for some $\gamma$ in N and $\delta$ in M. Note that in both L and L* the elements are in nonincreasing order within each flower, as they are maximized with respect to M. Also recall that the automorphisms in N preserve the order of vertex numbers within the flowers, by virtue of the fact that we coloured the vertices in order of vertex number when we computed N. This means that we can take $\delta$ to be identity, and so L* = $\gamma$(L). This proves that L* = L, since L = max { $\gamma$(L) | $\gamma$ in N}.

In order to implement the condition L = max { $\gamma$(L) | $\gamma$ in M}, we don’t need to compute M explicitly. Instead, since M is generated by transpositions, it suffices that within each flower the elements are in decreasing order relative to vertex number. Using the ordering of elements that we have chosen, in the example we just need to enforce the inequalities element(1) ≥ element(2) ≥ element(3), element(6) ≥ element(10) and element(9) ≥ element(11). The program recursively assigns elements to vertices in order of vertex number and enforces these inequalities as they become active rather than at the end.

To implement the condition L = max { $\gamma$(L) | $\gamma$ in N}, we compute N using nauty and test that $\gamma$(L) $\le$ L for each $\gamma$ in N. This is efficient in practice because N is very small most of the time.

We can also partly enforce N by means of inequalities: since vertex 6 is the least vertex in a non-trivial orbit {6, 9} of N, we can assume element(6) ≥ element(9). This is not necessary but it gives a small time improvement.

As an example, C_7_H_14_N_2_O_7_ has 15,425,657,612 isomers. Using the factorisation Aut(G) = NM reduces the number of nontrivial groups processed by 58% and the maximum group size from 2592 to 72. The overall generation time is 18% less. In typical cases, the method provides about 10–40% reduction in cost.

### Third stage

After the assignment of elements to vertices is complete, the program moves to the next stage of selecting a bond multiplicity for each edge. This is the same type of problem as in the second stage. Instead of a list of elements for each vertex, we have a list of multiplicities for each edge. Instead of Aut(G), we use the subgroup of Aut(G) that preserves the element assignment. Otherwise M and N are defined as before. In the implementation, we don’t use nauty to compute N but instead filter the N subgroup from the second stage, rejecting those automorphisms which don’t preserve elements and converting the others to their action on the edges.

The constraints we have at this time are that for each atom the total number of incident bonds counting multiplicity must be at most the valence of the atom, and that the total of (valence—incident bonds) over all atoms must equal the desired number of hydrogen atoms. Once these constraints are satisfied, there is exactly one way to add hydrogens (though the program does not add them explicitly).

As an example, geng makes 534,493 unlabelled simple graphs in 1.3 s for Lysopine C_9_H_18_N_2_O_4_. For these graphs, the second stage subgroup N is trivial 58% of the time and never larger than 72. Assignment of elements to vertices produces 3,012,069,151 vertex-labelled graphs in 90s.The N subgroup for the third stage is trivial 98% of the time and never larger than 24. Finally, the assignment of bond multiplicities produces 5,979,199,394 completed molecules in an additional 100 s.

As demonstrated by our examples, surge can generate molecular structures very quickly, allowing for the inspection of extremely large sets of isomers. The generation speed is several times faster than even the fastest output format (SMILES). On the other hand, any particular application will likely have stronger restrictions on the structure than just a molecular formula. For example, some substructures may make the molecule unstable or give it chemical properties undesirable in the application. Or, experimental investigation of an unknown compound may have determined some features of the structure, so that only molecules with those features are of interest.

For these reasons, surge provides a number of filters to limit the output. The 3-stage generation method allows some of them to be implemented almost for free, and all of them are much more efficient than filtering the output through an external program. For example, restrictions on the number of short rings and the planarity of the molecule can be enforced at Stage 1. Surge also provides some "badlists" of forbidden substructures (many of them inspired by the corresponding feature of MOLGEN).

The open-source nature of surge allows for a more advanced feature. By writing small code snippets, the user can insert custom filters into any of the three stages, and also perform such tasks as adding extra elements and command-line options. Several worked examples are provided with the program.

## Results

Surge is available under a liberal open-source License (Apache 2.0) on GitHub at https://structuregenerator.github.io as well as from https://users.cecs.anu.edu.au/~bdm/surge/.

The system can be built with the standard Unix Configure/Make scheme and the resulting stand-alone executable is then run from the command line. By default, surge generates all constitutional isomers of a given molecular formula. Surge can write output in either SDfile [26] or SMILES [27] format. SMILES output is produced very efficiently by constructing a template for each simple graph at the first stage, so that only atom types and bond multiplicity must be filled in before output.

**Fig. 5:**
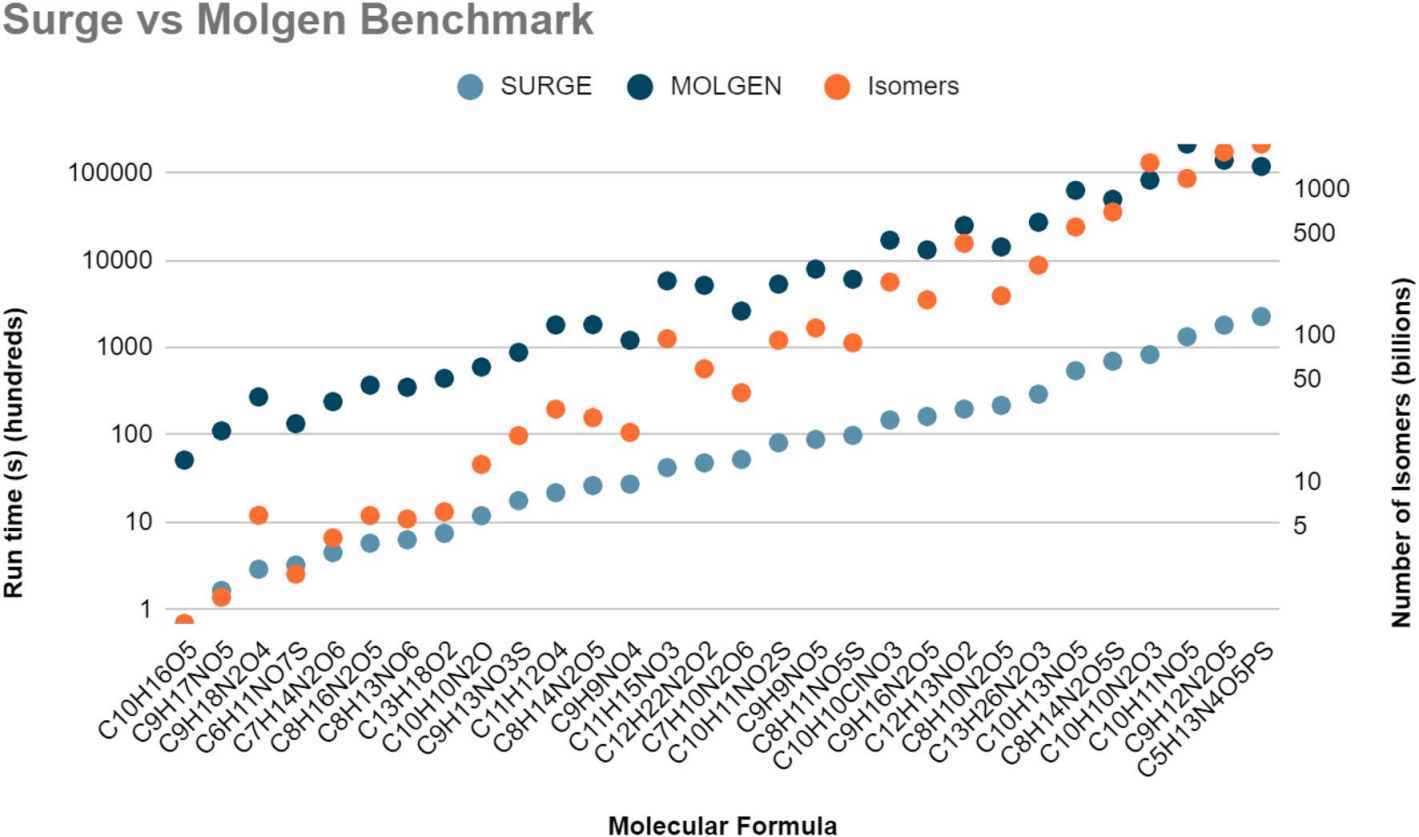
Comparison of the run times of surge v1.0 vs MOLGEN 5.0 for long-running molecular formulae from selected natural products, plotted on a logarithmic time scale. In the majority of cases, MOLGEN terminated at a built-in limit of 2^31^–1 structures. Reported computation times were linearly extrapolated based on the MOLGEN timing for 2^31^–1 structures and the actual number of isomers reported by surge

We benchmarked surge with the set of molecular formulae given in Table 1. Since our motivation for developing structure generators is for the generation of large molecules, Table 1 consists of natural products, randomly selected from the natural products database COCONUT [22]. For the list of molecular formulae, surge outperformed MOLGEN by orders of magnitude (Fig. 5) and MOLGEN terminated at a built-in limit of 2^31^–1 structures. Reported computation times were linearly extrapolated based on the MOLGEN timing for 2^31^–1 structures and the actual number of isomers reported by surge. Note that surge generates between 7 and 22 million molecules per second for all of these examples.

**Fig. 6:**
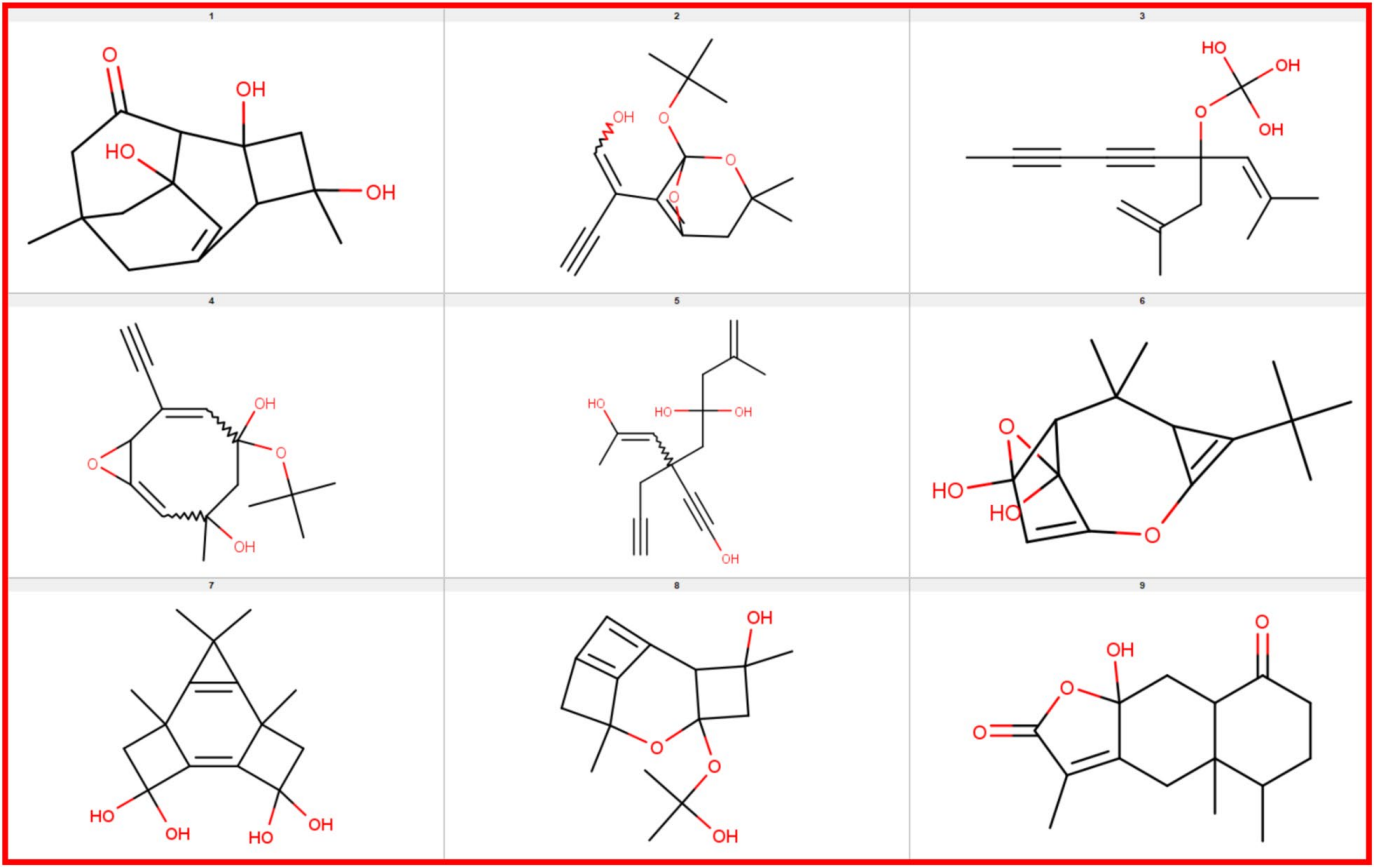
Nine example isomers of the natural product, Istanbulin A with the molecular formula C_15_H_20_O_4_. The molecular structure of Istanbulin A is given in the 9th entry in the above illustration

Surge has a tiny memory footprint irrespective of the molecule size or the number of isomers. All of the examples in this paper run in at most 5 MB of RAM on Linux (Fig. 6).

For randomly selected 10 molecular formulae, 4 options of surge were tested and results are given in Table 2. These options are.

-p0:1 At most one cycle of length 5
-P The molecule is planar
-B5 No atom has two double bonds and otherwise only hydrogen neighbours
-B9 No atom lies on more than one cycle of length 3 or 4.

### Limitations

Release 1.0 of surge does not perform a Hückel aromaticity test and therefore generates duplicate structures for Kekulé versions of aromatic rings that are graph-theoretically different. Benchmarking against MOLGEN 5.0 was therefore performed with the -noaromaticity switch of MOLGEN.

## Conclusion

We have presented surge, a structure generator for constitutional isomers based on the canonical generation path method. To the best of our knowledge, surge is the fastest chemical structure generator available. A number of badlist options are available to avoid the generation of potentially unlikely structures. Current limitations include the lack of an aromaticity detection. Surge is hosted as an open-source package on GitHub, inviting the scientific community to use and extend it. Surge offers a plug-in mechanism for community-driven extensions. Plugins can hook into the various stages of the surge generation process, thereby offering efficient means to prune the generation tree.

## Data Availability

Project name: surge Project home page: https://structuregenerator.github.io Operating system(s): Platform independent Programming language: C License: Apache 2.0
